# Psychosocial Distress as a Factor in Patients With Cancer Seeking Support: A Hermeneutic Study

**Published:** 2017-11-01

**Authors:** Marjan Mardani-Hamooleh, Haydeh Heidari

**Affiliations:** Department of Nursing, Iran University of Medical Sciences, Tehran, Iran, and Department of Nursing, Modeling in Health Research Center, Shahrekord University of Medical Sciences, Shahrekord, Iran

Cancer is one of the main causes of human death in the world, but its mortality rates have been in continual decline for the past 20 years (Siegel, Miller, & Jemal, 2015). It is a growing problem in Middle Eastern countries (Daher, 2011). In Iran, an ancient country in the Middle East (Borimnejad, Mardani-Hamooleh, Seyedfatemi, & Tahmasebi, 2014), cancer is the third most common cause of death, after heart disease and traffic accidents. The incidence of cancer in Iran is anticipated to be around 48 to 112 and 51 to 144 cases per year per million people for women and men, respectively (Seyedfatemi, Borimnejad, Mardani-Hamooleh, & Tahmasebi, 2014).

As the incidence of new cases of cancer increases every year, breaking bad news to patients is very important. Bad news is described as any piece of information that could potentially be directed to negatively change a patient’s expectations, ideas, feelings, or outlook (Salem & Salem, 2013). Breaking bad news to patients with cancer is a delicate and challenging task for most health-care providers (Eng, Yaakup, Shah, Jaffar, & Omar, 2012). Bad news about cancer creates pain for patients (Zebrack, Chesler, & Kaplan, 2010). It is unexpected and often may come as a shock (Yoo, Levine, Aviv, Ewing, & Au, 2010). In this regard, despite efforts by family members to conceal cancer diagnoses from patients, the majority of patients discovered the diagnosis of their own accord (Wang, Guo, Peng, Su, & Chen, 2011).

Breaking bad news to patients with cancer is diverse across different cultures (Table 1). Nondisclosure is the norm for Iranian people. However, health-care workers often want to tell patients of their diagnosis but worry that breaking bad news could evoke fear and anxiety for patients. In fact, it is difficult for health-care workers to predict cancer patients’ responses following disclosure. Therefore, a significant percent of cancer patients in many Mediterranean countries, such as Iran, are not made aware of their diagnosis, and many health-care workers prefer to disclose the cancer diagnosis directly to patients’ family members. However, the clinical experiences of the researchers show that family members rarely transfer this information to patients, and frequently prevent the disclosure of the cancer diagnosis. 

**Table 1:**
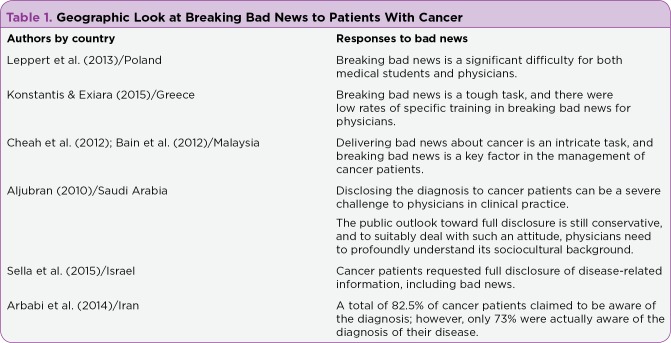
Geographic Look at Breaking Bad News to Patients With Cancer

**Abstract**

Unfortunately, formal education in breaking bad news is not currently incorporated in the medical curriculum in the majority of developing countries (Salem & Salem, 2013). In addition, while reviewing the medical literature, we found only a few qualitative studies on cancer patients’ hearing bad news within the context of Iranian culture. Due to cultural variations, perceptions of affected Iranian cancer patients may be different from those of individuals previously studied. It is therefore suitable to perform a qualitative study in this population. The aim of this study was to explore the lived experiences of Iranian cancer patients regarding hearing bad news.

## METHODS AND MATERIALS

**Design**

This qualitative research was conducted via a hermeneutic phenomenologic approach. It helps us to recognize the meaning of "being in the world." In other words, the results of this hermeneutic study provide perspective on the meaning embedded in lived experiences. Also, the nature of meaning in life experiences can be considered in depth (Heidegger, 1962). Therefore, contributing to hearing bad news is a way of "being in the world." In addition, our approach in this study allows cancer patients to focus on their lived experiences through an explanation of their individual experiences of hearing bad news.

**Study Context and Participants**

The study was performed in the cancer wards of two teaching hospitals specializing in the treatment of cancer patients in Tehran, Iran. These hospitals consist of cancer wards and outpatient clinics and serve as referral centers for cancer patients in Tehran.

After the oncology nurses identified the eligible informants, we invited 14 cancer patients to participate in the study. Participants were recruited into this study until data saturation was reached with 11 patients. Patients were recruited through the hospital inpatient wards and outpatient clinics. The sample size of phenomenologic studies is recommended by Creswell (2007) to be between 5 and 25 persons. Therefore, 11 patients took part: 6 females and 5 males. Their ages ranged from 33 to 54 years. Seven of the patients were married. The patients’ level of study ranged from elementary school to a bachelor’s degree. as for the type of cancer, four persons had breast cancer, three had colorectal cancer, two had hematologic cancer, one had gynecologic cancer, and one had prostate cancer. The minimum time from the diagnosis of disease was 3 months, and the maximum time was 4 years. Patients included in this study were aware of their diagnosis.

**Ethics**

The ethics committee of Tehran University of Medical Sciences approved the study. The data collection was carried out after obtaining a signed informed consent form from the participants. They were informed that they had the right to withdraw from the study at any time. They were assured that their answers would remain confidential and that their identity would not be revealed in any stage of the study.

**Data Collection**

Face-to-face, semistructured individual interviews lasting around 40 to 55 minutes were held in convenient quiet locations in the cancer wards. Each participant was interviewed once, for a total of 11 interviews. Over 4 months, patients were interviewed by the second author at their centers of treatment. The participants were interviewed alone, an interview guide was utilized, and the interviews were audio recorded.

The interviews were performed in the Persian language by the second author. Those parts of the interviews that were related to this article were translated into English by a professional translator, and then the English version was converted back into Persian for verification by the first author.

The patients were requested to explain their lived experiences in their own words to the question of "What is the meaning of hearing bad news?" After the participants responded to the question, more questions were asked to gain richer data, such as "Would you explain more about this?", "What is the meaning of that idea?", and "Could you please give an example to help us suitably comprehend your point of view?"

**Data Analysis**

Data gathering and analysis occurred in parallel. Teamwork was used in this study to analyze the data. Therefore, to analyze and achieve a better understanding and interpretation of the lived experiences of the participants, we used the seven-stage process of data analysis (Diekelmann, Allen, & Tanner, 1989):

*Stage 1:* Each interview text was initially checked as a whole to gain a general understanding.

*Stages 2 and 3:* Probable common meaning units were then identified, using extracts to support the interpretation. The authors repeatedly listened to the tape recordings to extract the true meaning of the data.

*Stage 4:* The authors evaluated their interpretations for similarities and differences, gaining more clarification and agreement by revisiting the primary text.

*Stage 5:* All texts were then revised to confirm emergent themes and subthemes. Next, the emerging themes and subthemes were classified by the authors.

*Stage 6:* A constitutive pattern that indicated the relationship between the themes and subthemes across all texts was recognized.

*Stage 7:* The research team created a final report, including quotes that were permitted for confirmation by the reader.

The trustworthiness of the study is supported by four criteria: credibility, dependability, conformability, and transferability (Lincoln & Guba, 1985). To achieve credibility, opinions of the research group were used in the procedure of interviews and data analysis. Interview texts, extracted meaning units, as well as themes and subthemes were discussed by some patients and two persons with a PhD in nursing. To determine data dependability, the views of an external viewer, who was a researcher familiar with phenomenologic study and not a member of the research team, were used. There was an agreement on the findings. To obtain conformability, all the actions were recorded, and a report was prepared on the research progression. To obtain data transferability, data collected from two participants outside of the study who had circumstances similar to those of study patients were argued and verified.

## RESULTS

Cancer patients’ lived experiences of hearing bad news were grouped into two main themes: distress and seeking support. These themes reflected the meaning of hearing bad news by cancer patients. The constitutive pattern of the study was psychosocial distress as a factor for patients with cancer seeking support. The study themes and quotations from patients are listed in Table 2.

**Table 2:**
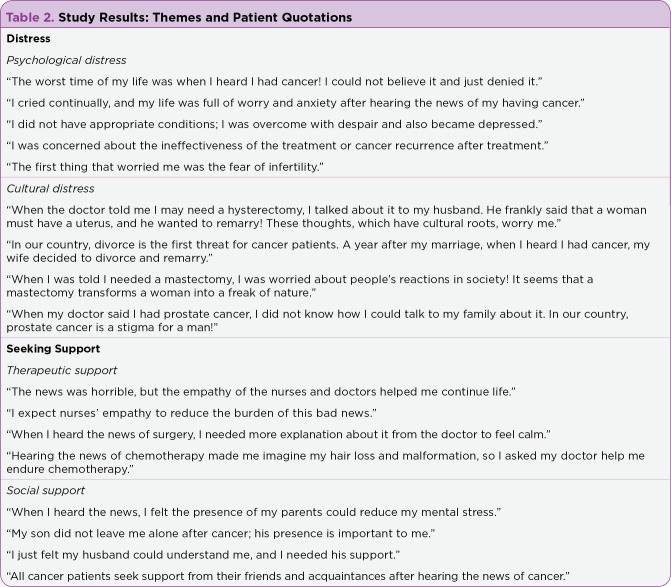
Study Results: Themes and Patient Quotations

**Distress**

*Psychological Distress:* The participants experienced denial, anxiety, despair, and depression after hearing the news of a cancer diagnosis, which led to their psychological distress. An effective factor that resulted in psychological distress in cancer patients after hearing the bad news of a cancer diagnosis was their worry about treatment results and recurrence of cancer. Moreover, the fear of infertility could be counted as a consequence of hearing the news of cancer.

*Cultural Distress:* The participants experienced cultural distress after hearing the news of cancer. They believed it to be rooted in false cultural beliefs of the society in a way that female cancer patients were concerned about their husbands remarrying. The cancer patients expressed that hearing the news of cancer may lead to divorce. This outcome of cancer, which is common in our society, was very unpleasant for these patients. Women experienced distress after mastectomy, which resulted from aversive reactions of people and their false beliefs. According to the experiences of participants, in Iranian culture, hearing the bad news and revealing it to their family made them feel ashamed. Considering cancer as a stigmatized disease demonstrates cultural distress.

**Seeking Support**

*Therapeutic Support:* After hearing the news of cancer, patients sought therapeutic support from nurses and doctors, which was associated with better life adaptation for the patients. In addition, cancer patients sought empathy from nurses and information support from doctors to decrease their cultural distress.

*Social Support:* After hearing bad news, the patients sought a range of social support from family, friends, and acquaintances to reduce mental distress. Iranian cancer patients experienced psychosocial and cultural distress after hearing the news of cancer. Subsequently, the patients sought therapeutic and social support from health-care providers, family, and friends. Unfortunately, false cultural beliefs often lead to some unpleasant consequences for these patients, including divorce, the stigma associated with having cancer, people’s aversive reactions, and embarrassment.

## DISCUSSION

The findings of this study showed that cancer patients sought therapeutic and social support to alleviate the psychosocial and cultural distress of hearing bad news. The participants experienced a range of mental distress after hearing the bad news. Many factors created psychosocial distress, including decline, denial, anxiety, despair, depression, fear of treatment ineffectiveness or recurrence of cancer, and infertility.

The results of this study are consistent with research conducted in Norway, which showed cancer patients suffered from severe tension due to their disease (Heyn, Finset, Eide, & Ruland, 2013). Moreover, research in Australia demonstrated that potential fertility was a major concern for female cancer patients (Penrose, Beatty, Mattiske, & Koczwara, 2012). Also, fear of recurrence was counted as a key consideration in patients with cancer in a study from Scotland, which determined the importance of offering support to these patients (Hubbard, Venning, Walker, Scanlon, & Kyle, 2015).

In addition, Iranian cancer patients encountered an extensive range of cultural distresses due to false social beliefs, such as a femininity fault following mastectomy and hysterectomy, divorce and spouse remarriage, stigma of cancer, and feeling embarrassment over talking about their disease. The results of one study suggested the main concern of female cancer patients in Bahrain was their spouse’s remarriage, which is rooted in cultural challenges (Jassim & Whitford, 2014). Consistent with these findings were the results of a study suggesting Jordanian women perceive cancer as a stigmatized disease (Alqaissi & Dickerson, 2010).

The study patients sought therapeutic and social support to overcome distress caused by cancer. Some studies performed in Sweden, Canada, Japan, and Iran demonstrated the necessity of offering support to cancer patients (Malmström, Ivarsson, Johansson, & Klefsgård, 2013; Pedersen, Hack, McClement, & Taylor-Brown, 2014; Yoshida et al., 2010).

The participants sought therapeutic and emotional support from health-care providers, especially nurses and doctors. They also sought doctors’ informational support over surgery and chemotherapy. In fact, Chinese cancer patients who were US residents underscored the importance of doctors’ empathy at the time of cancer diagnosis (Wang et al., 2013). Furthermore, a study carried out in Malaysia indicated that cancer patients who were seeking doctors’ support received it while hearing the bad news (Eng et al., 2012).

Furthermore, the study patients also sought social support from family, friends, and acquaintances to lessen their distress after hearing bad news. Consistent with this practice, results of a study conducted in the United States showed that cancer patients searched for social support (Yoo et al., 2010). The results of another study in France demonstrated the role of social support in decreasing anxiety and depression in cancer patients (Lafaye et al., 2014).

Generally, although cancer patients desired social and therapeutic support to decrease their distress caused by hearing the bad news of a cancer diagnosis, cultural factors often strengthened these distresses. Consequently, it seems that decreasing the effectiveness of cultural factors on hearing such bad news in Iran may lead to reducing this cultural distress, which would require extensive cultural interventions. Regrettably, the cultural ideas about cancer are still present among Iranian people, indicating cancer remains a taboo subject. In Iran, talking about cancer is very challenging. There is a general idea in Iranian culture that a cancer diagnosis is considered a symbol of death (Heidari & Mardani-Hamooleh, 2016). In addition, the Iranian culture views cancer as a stigmatized disease to be concealed from society (Mardani-Hamooleh & Heidari, 2016).

Cultural and supportive interventions to facilitate positive change might decrease suffering and enhance positive outcomes for cancer patients. Culture and support services that help people deal with the impact of cancer on life after a cancer diagnosis and treatment must be made available. In fact, since the results of our study showed that cancer patients are confronted with cultural distresses with regard to hearing bad news, we may decrease these distresses by producing educational programs on television.

This study had some limitations as well. Patients were not chosen based on a certain type or stage of cancer. For other researchers, we suggest breaking bad news in new cases of cancer patients. The small sample size and the nature of the study limited our ability to generalize the findings. However, as with all qualitative studies, the results were not intended to be generalized. Nevertheless, out study findings add to the body of knowledge in this area.

## IMPLICATIONS FOR ADVANCED PRACTITIONERS

Our study findings have important clinical implications. Health-care providers should attend to patients’ conditions as well as the challenges of the cultural, physical, emotional, and social sequelae of hearing bad news in cancer patients. Our findings suggest that cultural supportive services are needed for these patients in Iran. Further qualitative studies are obviously required to more completely comprehend the impact of hearing bad news on Iranian cancer patients. In fact, qualitative studies that provide a deeper understanding of this topic, specifically those related to the problems associated with cultural stigma, would provide significant insight.

**Acknowledgments**

The authors are grateful to all study participants who were willing to share their time with us.
